# Acknowledgement to Reviewers of *Animals* in 2019

**DOI:** 10.3390/ani10020183

**Published:** 2020-01-21

**Authors:** Animals Editorial Office

**Affiliations:** MDPI, St. Alban-Anlage 66, 4052 Basel, Switzerland

The editorial team greatly appreciates the reviewers who have dedicated their considerable time and expertise to the journal’s rigorous editorial process over the past 12 months, regardless of whether the papers are finally published or not. In 2019, a total of 1219 papers were published in the journal, with a median time to first decision of 41 days and a median time from submission to publication of 22 days. The editors would like to express their sincere gratitude to the following reviewers for their generous contribution in 2019:
A.K. Salama, AhmedLi, YinA.T. Borojeni, AzadehLi, YuzhiAaltola, ElisaLi, YuzhiAbd El-Hack, MohamedLi, ZhuanjianAbdallah Abdelmohsen, MohamedLiermann, WendyAbdeen, AhmedLillo, IsidoroAbdel-Mageed, Ahmad M.Lim, Kyu-SangAbdollahi-Arpanahi, RostamLima Rodrigues, Jhennyfer AlineAbecia, LeticiaLin, HaiAbegão, LuisLin, Hui-ChuAbeni, FabioLin, Yu-HungAbo-Ismail, MohammedLind, VibekeAbuelo, AngelLindholm-Perry, Amanda KAbuelo, ÁngelLindner, Arno E.Acharya, MohanLinnell, JohnAdamczyk, KrzysztofLipiński, KrzysztofAdams Progar, AmberListrat, AnneAdams, SeiduLittin, KateAdams, Vicki J.Littlewood, KatAdamski, MarekLiu, BangAdamski, MarekLiu, HaiboAdler, Steffen A.Liu, HongyunAdrian, DerekLiu, JinxinAdunyah, Samuel E.Liu, JundiAggarwal, AbhishekLiu, KaiAgulheiro Santos, Ana CristinaLiu, QiangAhlawat, SonikaLiu, XiaoleiAkins, MattLiu, YanhongAkter, Yeasmin AraLivingston, Kimberly AnnAlagwany, MahmoudLlonch, PolAlarcón López, Francisco JavierLloyd, JaniceAlbano, MarcoLockwood, RandallAlbarella, SaraLokhorst, KeesAlberghina, DanielaLombardi, JasonAlbert, EliseLombardi, PietroAlbertini, MariangelaLoncoman, CarlosAlbrecht, ElkeLong, NathanAl-Bulushi, SamirLoor, Juan J.Aldrich, Charles GregoryLopes, Ana Patrícia AntunesAleri, JoshuaLópez Albors, OctavioAlexander, Brenda M.López Vergé, SergiAlexandre, Pâmela AlmeidaLopez, IgnacioAlgers, AnneLópez, SecundinoAli, AliLopez-Bote, ClementeAlibhai, Hatim I.K.López-Cortegano, EugenioAlić, AmerLopez-Salesansky, NoeliaAlkhtib, AshrafLopreiato, VincenzoAllain, DanielLorenzo, Jose M.Allard, StephanieLouton, HelenAllen, AndrewLouton, HelenAllen, DanielLove, GordonAlleva, EnricoLu, EnliAlleva, EnricoLu, MingzhouAl-Marzooqi, WaleedLucas-Hahn, AndreaAlonge, SalvatoreLucchesi, PaulaAlonso, Marta LópezLucci, CarolinaAl-Sagheer, AdhamLuciana, RossiAltomonte, IolandaLuck, LenaÁlvarez-Rodríguez, JavierLuddi, AliceAlves, RômuloLudwiczak, AgnieszkaAlves, Susana P.Luise, DianaAmado, SandraLuisetto, RobertoAmadori, MassimoLumineau, SophieAmano, TomokoLunesu, MondinaAmendola, LuciaLunge, Vagner RicardoAmetaj, BurimLuo, HailingAm-in, NuttheeLuo, YuzhuAmlund, HeidiLupo, Karen D.An, Dong-JunLuron, IsabelleAnastasiadou, MariaLürzel, StephanieAndersen-Ranberg, Emilie U.Luvoni, Gaia CeciliaAnderson, JamesLynch, BridgetAnderson, NicholeLynch, TarahAnderson, Robin CM. McDonnell, SueAndrade-Montemayor, Héctor MarioMa, YibaoAndrás, GáspárdyMa, YongxiAndrea, VitaliMa, YuehuiAndrés, SoniaMacciotta, Nicolo’ Pietro PaoloAndueza, DonatoMacedo Barragán, Rafael JulioAnglani, FrancaMacNamara, MaureenAngrecka, SabinaMacrae, Alastair I.Annibalini, GiosuèMaddock, RobertAnnicchiarico, GiovanniMadeira De Carvalho, Luis ManuelAnraku, MakotoMadkour, AichaAntonini, MarcoMaertens, LucAparicio, Miguel A.Maes, DominiekApperson, K. DeniseMagalhães Sant’Ana, ManuelArana, AnaMagalhães-Sant’Ana, ManuelArczewska-Włosek, AnnaMaggiolino, AristideArgente, María-JoséMagistrali, Chiara FrancescaArias I., Rodrigo A.Magnabosco, DiogoArias Sanchez, RamonMahabir, EstherArmand, Anne-SophieMahony, Timothy JohnArnevik, Espen AjoMahsoub, HassanArney, DavidMaia, MargaridaArnold, Bruce BaerMainau Brunso, EvaArranz Santos, Juan-JoseMaiorano, GiuseppeArrazola, AitorMajecka, KatarzynaArriaga-Jordan, CarlosMajewska, MałgorzataArtegoitia, Virginia M.Makecha, RadhikaArts, KoenMakowska, I. JoannaAryal, AchyutMalavasi, RacheleAsai-Coakwell, MikaMalchow, JuliaAsher, LucyMalkemper, PascalAstiz, SusanaMallem, Mohamed YassineAtes, SerkanMalmkvist, JensAttia, YoussefMałopolska, MartynaAttia, Youssef A.Maltez, LucasAtzori, Alberto S.Manafiazar, GhaderAureli, FillipoManchester, AlisonAurich, ChristineMancinelli, AliceAveros, XavierMancini, SimoneAverós, XavierMancini, SimoneAvondo, MarcellaMandel, RoiAwosile, BabafelaManin, AurélieAzain, MichaelManjarin, RodrigoAzarpajouh, SamanehMann, SabineBaars, TonManor, JuliaBacci, Maria LauraManuelian, CarmenBachmann, MartinMao, DaganBaciadonna, LuigiMao, YongjiangBackus, Brittany L.Maple, TerryBacon, HeatherMarashdeh, OmarAlBadiani, AnnaMarcet-Rius, MíriamBadino, PaolaMarchina, ChiaraBagasra, AnisahMarcondes, Marcos InácioBagnicka, EmiliaMarechal, LaetitiaBai, ShipingMargerison, JeanBaidoo, SamMargulis, Sue W.Bailey, R. HartMarín-García, PilarBaird, BonnieMarini, DanilaBajwa, NaeemMariti, ChiaraBakaloudis, DimitrisMarletta, DonataBaker, LivMarock, BryceBalcells, JoaquimMaros, KatalinBalčiauskienė, LaimaMaroto-Molina, FranciscoBaldi, GiuliaMarounek, MilanBaldinger, LisaMarques, MarianaBaldwin, Roger A.Marsh, Antoinette E.Ballini, AndreaMarsico, GiuseppeBanaszewska, DorotaMartelli, GiovannaBanda, Liveness JessicaMarth, ChristinaBandara, Rathnayaka AmilaMartin, AdamBandara, Rathnayaka Mudiyanselage Amila SubhashinieMartin, Angela K.Bandoli, FrancescaMartin, JessicaBani, PaoloMartinelli, DarioBaragli, PaoloMartínez Marín, Andrés LuisBarba Recreo, MartaMartín-Hidalgo, DavidBarbas, João PedroMartino, GiuseppeBarbato, MarioMartins, JPBarbieri, SaraMartins, Maurício LaterçaBard, Kim A.Martins-Bessa, AnaBarekatain, RezaMarzocco, StefaniaBarłowska, JoannaMason, FedericoBarnard, ShanisMason, GeorgiaBarranco, IsabelMastrangelo, SalvatoreBarrón-Ortiz, Christina I.Masucci, FeliciaBarstow, AmyMather, JenniferBarszcz, MarcinMátis, GáborBartolome, EsterMatsubara, HajimeBartolomé, JordiMatsumoto, HirokazuBarton, LudekMatthews, JamesBasini, GiuseppinaMattiello, SilvanaBassetto, CesarMattioli, SimonaBassi, IvanaMatusevičius, PauliusBatargias, CostasMaurer, PatricBatkowska, JustynaMavangira, VengaiBattelli, GiovannaMawson, PeterBaumans, VeraMawson, Peter R.Baumrucker, CraigMaxwell, HerrisBawden, SimonMayer, IanBazer, FullerMazon-Suástegui, José ManuelBazzano, MarilenaMazzola, Silvia MichelaBazzino Ferreri, Gaston A.McBride, AnneBeaulieu, DeniseMcClelland, GrantBeausoleil, NgaioMcCobb, EmilyBeck, AlanMcCowan, BrendaBeck, Paul AMcCrackin, Mary AnnBédécarrats, GrégoyMcDonald, RobbieBeerda, BonneMcEwan, NeilBeggs, David SMcFarlane, JamesBeigh, Yasir AfzalMcGee, MarkBelenguer, ÁlvaroMcGill, JodiBelghit, IkramMcGlone, JohnBellagamba, FedericaMcGregor, HughBenbrook, CharlesMcGuire, BettyBenjamin, MadonnaMcHugh, NoirinBenjamin, MadonnaMcKeever, Kenneth H.Benmerzouga, ImaanMcLean, AmyBennet, E.D.McLennan, KristaBennett, DarinMcMichael, Maureen A.Benowitz, Kyle MMcMillan, FrankBenson, EricMcQuade, RachelBentosela, MarianaME, CarvalhoBenvenutti, MarceloMeadus, William JonBerg, CharlotteMeagher, RebeccaBergamaschi, MatteoMedger, KatarinaBerge, Anna CatharinaMedica, PietroBerger, AnneMedrano, Juan HernandezBergfelt, DonMeehan, CherylBergkvist, GuraaMeeker, David L.Bergmann, ShanaMei, JieBergschmidt, AngelaMeijer, EllenBerman, AmielMejdell, CecilieBernhard, WolfgangMekuchi, MiyukiBernhardt, HeinzMellersh, CathrynBernstein, MarkMellor, David J.Berny, PhilippeMenchaca, AlejoBerrocoso, Julio Francisco DíazMenchetti, LauraBertella, GiovannaMendonça, TiagoBertelloni, FabrizioMendyk, RobertBerteselli, Greta VeronicaMeneguz, MarcoBertin, Francois-ReneMeneguz, Pier GiuseppeBertocchi, MartinaMeng, HeBertolotti, LuigiMenna, Lucia FrancescaBertoni, GiuseppeMenoyo, DavidBessei, WernerMenzies, PaulaBestman, MoniqueMercadante, Vitor Rodrigues GomesBhak, JongMercati, FrancescaBhatti, Muhammad AzherMercer, JennyBiasato, IlariaMerkies, KatrinaBickerton, EricaMerrill, EvelynBielas, WiesławMesquita, JoãoBienboire Frosini, CecileMetrione, LaraBiffani, StefanoMetzger, JuliaBijak, MichałMeyer, LeithBirke, LyndaMeyer, Robert E.Birke, LyndaMeza, CesarBirolo, MarcoMezzetti, MatteoBiscarini, FilippoMiao, XiangyangBissonnette, NathalieMiddleton, John R.Bittante, GiovanniMielenz, ManfredBittencourt, Daniela Matias De CarvalhoMigliorini, Marcos J.Bjalobok, FaithMiguel-Pacheco, Giuliana G.Björkman, StefanMihalca, Andrei DanielBlackie, NicolaMihina, ŠtefanBland, IanMikko, SofiaBlasdell, KimMilburn, JoshBlatchford, Richard A.Milczarek, AnnaBlavi, LaiaMiller, Amy L.Bleach, EmmaMiller, GemmaBlikslager, AnthonyMiller, KellyBlokhuis, HarryMiller, Stephen P.Bohrer, Benjamin M.Min, ByengryelBokkers, EddieMinervino, Antonio Humberto H.Bolwell, CharlotteMinnie, LiaanBombail, VincentMiranda, CarlaBombardi, CristianoMiranda, MartaBombieri, GiuliaMiró, FranciscoBonanno, AdrianaMiro, JordiBonati, BeatriceMiron, JoshuahBonato, MaudMiron, JoshuahBonfatti, ValentinaMiscioscia, ErinBongiorni, SilviaMishina, TappeiBonnet, Alejandro SuarezMiskulin, IvanBonnet, XavierMitchell, KatharynBonos, EleftheriosMiura, NaokiBoone, JohnMiura, TakeshiBooth, DavidMiyake, YoichiBordin, LucianaMo, DelinBorg, BertilMoate, Peter J.Bortoluzzi, CristianoMochizuki-Kobayashi, MarikoBoschetti, ElisaModina, Silvia C.Bosi, PaoloMoeller, Steven J.Böttger, ChristianMogodiniyai Kasmaei, KamyarBourassa, Dianna VMohan-Gibbons, HeatherBourneuf, EmmanuelleMolento, CarlaBoutinaud, MarionMolento, Carla Forte MaiolinoBouwman, AniekMollo Neto, MarioBovera, FulviaMonahan, FrankBovera, FulviaMonchatre-Leroy, ElodieBowen, JonMondaca, MarioBowen, MarkMongillo, PaoloBowker, B. C.Moniello, GiuseppeBowles, DavidMontaldo, HugoBoyd, R. DeanMontgomery, Tracy M.Bozzo, GiancarloMontillo, MartaBradshaw, PatrickMontoya Alonso, Jose AlbertoBran, José A.Moon, YuseokBranco, PauloMoons, ChristelBrandt, ChristianMoore, KarenBransfield, RobertMoore-Colyer, MerielBrauchle, MichaelMorais, Tiago G.Bravo, CarolinaMorales-Ramos, Juan A.Brearley, JackieMoran, Colm A.Breheny, MaryMordenti, AttilioBreitenbecher, KimberlyMoreira, Gabriel Costa MonteiroBremhorst, AnnikaMoretti, RiccardoBriefer Freymond, SabrinaMorgan, KathleenBright, TerriMorgan, NatalieBritti, DomenicoMorgan-Davies, ClaireBrockington, DanMori, EmilianoBrodzki, PiotrMorittu, Valeria MariaBroer, StefanMörlein, DanielBronzo, ValerioMornement, KateBroom, Leon J.Morris, PaulBroucek, JanMorris, TimBrown, Christopher LyonMorsy, TarekBrown, HiekeMorton, DavidBrown, JenniferMoschopoulou, EkateriniBrown, SarahMoshammer, HannsBrowning Jr., RichardMott, AlexanderBrščić, MartaMoula, NassimBrucks, DesireeMoura, Ana Silvia A. M. T.Brugger, DanielMoura, Marcelo TigreBrunet, FrédéricMoustsen, Vivi AarestrupBuccioni, AriannaMoya, DiegoBuchmann, AnneMT, SkowronskiBuckland, EmmaMuboko, NeverBuckley, FrankMukherjee, RajibBueno Dalto, DanyelMullan, SiobhanBugg, WillMüller, ArianeBuhr, Richard JeffMüller, CeciliaBuitenhuis, Albert JohannesMunday, JohnBulc, MichałMuñoz, AnaBuonavoglia, CanioMunoz, CarolinaBurbi, SaraMunsterhjelm, CamillaBurghardt, GordonMurakami, HitoshiBurke, JenniferMurawska, DariaBurkey, Thomas E.Muroya, SusumuBush, Stephen JMurray Stewart, TracyBushby, Philip A.Murugesan, G. RajButler, DeborahMusch, MarkByosiere, SarahMusco, NadiaByosiere, Sarah-ElizabethMusk, Gabrielle C.Cabrera, Victor E.Musto, MauroCacciotto, CarlaMuszyński, SiemowitCaffo, LeonardoNääs, IrenilzaCahaner, AvigdorNagai, KouheiCaixeta, LucianoNagasawa, KazueCajarville, CeciliaNagashima, HitoshiCakebread, PeterNagashima, Jennifer B.Calabrò, SerenaNaguib, Mahmoud MohamedCaldas, Lucio AyresNahashon, SamuelCallens, BenedicteNainiene, RasaCalvo, JorgeNakajima, IkuyoCâmara, Diogo RibeiroNalon, ElenaCambier, CaroleNalon, ElenaCamerlink, IreneNanni Costa, LeonardoCameron, LornaNannoni, EleonoraCamin, FedericaNapierala, MartaCammack, Jonathan A.Natalello, AntonioCampbell, DanaNatoli, EugeniaCampelo, DanielNaval-Sanchez, MarinaCampos, Claudia M.Navas González, Francisco JavierCandek-Potokar, MetaNawaz, Dr. MuhammadCanibe, NuriaNawaz, Muhammad ZeeshanCannas, AntonelloNayeri, ShadiCantor, Melissa C.Neave, HeatherCao, BinyunNeglia, GianlucaCao, DeborahNery, JoanaCaparros-Martin, JoséNeuditschko, MarkusCapillo, GioeleNeupane, MaheshCapion, NynneNewey, ScottCappai, Maria GraziaNg, ZenithsonCarabaño, María J.Nguyen, TH (Huyen)Cardona, CarolNicholas, FrankCardoso, Clarissa SilvaNiciura, SimoneCareem, FaizalNicol, A.Caria, MariaNiedziałkowska, MagdalenaCarlberg, CarstenNielsen, BrianCarmen, MejíaNielsen, Martin K.Carney, Hazel C.Nielsen, Tina S.Caroprese, MariangelaNiero, GiovanniCarr, ChadNiesen, PeterCarracha, Rui MiguelNieuwland, JoachimCarral Llamazares, José ManuelNilforooshan, Mohammad AliCarretón, ElenaNinomiya, ShigeruCarroll, Grace A.Nishida, TakehiroCarter, AnneNishimura, RyoCarter, CraigNishizawa, BungoCarter, JacobyNiu, DongyanCarvalho, António PauloNiu, MutianCassandro, MartinoNixon, JaneCassar, GlenNizza, AntoninoCastaldi, AlessandraNoakes, DavidCastellini, CesareNobis, NathanCastiglioni, BiancaNocillado, JosephineCastiglioni, BiancaNoda, Juan J.Castro Esteves, Pedro JoséNogueira, Daniel M.Castro-Montoya, JoaquinNogueira, Guilherme De PaulaCaulfield, MalcolmNoh, Hyun JiCeacero, FranciscoNomura, KazuharuCeballos, Maria CamilaNonneman, Danny J.Celi, PietroNormando, SimonaCerino, StefaniaNorring, MariannaChaber, Anne-LiseNorris, Jacqueline M.Chacón, Ruy D.Nout-lomas, YvetteChadwin, RobinNovák, KarelChae, Byung-JoNovellas Torroja, RosaChai, JinNowacka-Woszuk, JoannaChai, LilongNowakowicz-Dębek, BożenaChandler, Cynthia KayNowotny, ManuelaChapple, Christopher KeyNuñez, Cassandra M VChapwanya, AspinasNuzzo, DomenicoCharmley, EdNzelu, ChukwunonsoChase, LarryOanh, Nguyen CongChatelain, MarionOberbauer, Anita M.Chatzis, ManolisObitsu, TaketoChaves, AlexandreOcepek, MarkoChe, LianqiangO’Connell, Timothy J.Chen, Chin-ShanO’Connor, CherylChen, ChongxiaoOczkowicz, MariaChen, DaqingOdemer, RichardChen, Jiann-ChuOdore, RosangelaChen, TianshengO’Driscoll, KeelinChen, XiaowuOgawa, ShinichiroCheng, HengweiOgi, AsahiCheng, JianboOgino, YukikoCheng, LinOgnik, KatarzynaCheng, Vincent Y-HOhh, Sang-jipChenier, TraceyOhta, MitsuakiChiang, Hsin-IOikonomou, GeorgeChidgey, KirstyOjima, NobuhikoChimienti, MariannaOkino, Cintia HiromiChimonyo, MichaelOkochi, HitoshiChincarini, MatteoOkpeku, MosesChiofalo, BiaginaOlesen, IngridCho, JongkiOliveira, Ronaldo LopesChoi, Yoon KyungOliveira-Filho, José P.Chon, Tae-SooOliver, MarkChou, KarenOliver, William ThomasChoudhuri, AvikOlivotto, IkeChow, SeinenOllivier, MorganeChristensen, Bruce W.Olsson, AnnaChristensen, HenrikOlukosi, Oluyinka A.Christopherson, PeterOpaliński, SebastianChu, MingxingOrczewska-Dudek, SylwiaChuanying, PanOrengo, JuanChud, TatianeO’Riain, JustinChulayo, Amanda YuccaOrkusz, AgnieszkaChung, Wen-SungOrlandella, Bianca MariaCiccarelli, MichelaOrozco-terWengel, PabloCieniewski-Bernard, CarolineOrr, BronwynCieślak, JakubOrtuño Casanova, JordiCieślińska, AnnaOsei-Amponsah, RichardČikoš, ŠtefanO’Shea, CormacCilia, GiovanniOsthaus, BrittaCiliberti, Maria GiovannaOtten, WinfriedCinnamon, YuvalOtto, JohnCinq-Mars, DanyOttoboni, MatteoCintas, LuisOughton, MatthewCitek, JindrichOwens, David W.Ciulu, MarcoOzella, LauraClark, BethPacelli, CorradoClark, CameronPadalino, BarbaraClark, EmilyPadalino, BarbaraClark, SherriePadalkar, MugdhaClauss, MarcusPaez, EzeClavenzani, PaoloPage, AllenClegg, IsabellaPage, StephenClegg, SimonPageat, PatrickCluff, KimPaillot, RomainCocchia, NatasciaPalazzo, MarisaCogger, NaomiPalma Sircili, MarceloColazo, Marcos G.Palme, RupertCollins, AngelaPalmerini, MariaCollinson, WendyPalsamy, PeriyasamyColombini, StefaniaPalus, KatarzynaColombo, Fabio MariaPan, GuohuiColpoys, JessicaPan, JinmingColumba, PietroPandelides, ZachariasComizzoli, PierrePandey, Atul KumarCone, John W.Panee, JunConnor, MelaniePaneru, BamConnor, MelaniePaolini, AlessandraContalbrigo, LauraPaolucci, MarinaContiero, BarbaraPapademas, PhotisContina, AndreaPapadopoulos, GeorgiosContreras, G.AndresPapet, IsabelleContreras-López, ManuelParanhos Da Costa, Mateus José RodriguesConway, WarrenParissi, Zoi M.Coombe, JoParkes, RebeccaCoppola, CristaParolini, MarcoCoppola, Crista L.Parr, SadieCorballis, MichaelParraguez, Víctor HugoCorbi, GraziamariaParrilla, InmaCorreddu, FabioPaschino, PietroCorreia Caeiro, CatiaPascual, JuanCorriero, AldoPasicka, EdytaCortes, OscarPassantino, AnnamariaCortes, OscarPassler, ThomasCortese, LauraPastore, CarloCortez-Romero, CesarPastorino, PaoloCosentino, CarloPathak, DhrubaCosta, DiogoPatra, Amlan KumarCosta, Manuela DallaPatt, AntoniaCottrell, JeremyPatterson, MandyCouceiro, Sheyla R.M.Patton, Robert A.Coulter, KendraPaudyal, SushilCove, MichaelPaul, Long ChengCowie, SarahPausch, HubertCozzi, BrunoPauselli, MarianoCozzi, CristinaPavlata, LeosCraig, Bruce A.Pawar, KamleshCraig, JessicaPawlak, AleksandraCrandell, KathleenPawlowski, LucjanCremonesi, PaolaPayne , AnnetteCrenshaw, ThomasPayne, StevenCrescimbene, LaraPazdro, RobertCrespo-Piazuelo, DanielPazzola, MicheleCrisá, AlessandraPebsworth, Paula A.Croft, David B.Pechova, AlenaCronin, Katherine A.Pecka-Kiełb, EwaCrosta, LorenzoPedersen, JanniCrovetto, Gianni MatteoPedersen, Lene JuulCrowley, SarahPedraza Chaverri, JoséCrowther, MathewPeggs, KayCrupi, RosaliaPeimbert, MarianaCui, HuanxianPeiretti, Pier GiorgioCukor, JanPeli, AngeloCullere, MarcoPellis, Sergio M.Cushman, Robert APeña-Espinoza, MiguelCutrignelli, Monica IsabellaPendry, PatriciaCyklis, PiotrPereira Lestayo, VíctorCzech, AnnaPerera, NipunaCzeglédi, LeventePeretti, VincenzoCzerniawska-Piątkowska, EwaPerez Ibarra, IreneCzopowicz, MichałPérez, José FranciscoCzopowicz, MichałPérez-Enciso, MiguelCzycholl, IrenaPérez-López, Antonio J.Da Borso, FrancescoPerkins, SarahDa Silva, Lorrayny GaloroPero, Maria ElenaDaetwyler, Hans D.Péron, FranckDahl, GeoffreyPerrucci, StefaniaDahlke, GarlandPesti, Gene M.Dahl-Pedersen, KirstinPetersen, Mette BisgaardDai, FrancescaPeterson, SamDai, XiaoxiaPetitte, JimDaigle, CourtneyPetrera, FrancescaDal Bosco, AlessandroPetropoulos, Spyridon A.Dale, RachelPetrovski, KiroDalla Villa, PaoloPezzuolo, AndreaDall’Aglio, CeciliaPfeuti, GuillaumeDalle Zotte, AntonellaPhysick-Sheard, P. W.Dalmau, AntoniPiantino Ferreira, Antonio JoséDalmau, AntoniPiao, XiangshuDalton, K. P.Piccolo, GiovanniDamasceno, Flavio A.Pickett, AndyDamaziak, KrzysztofPickova, JanaDanezis, GeorgePieramati, CamilloDang, RuihuaPierard, MarcD’Aniello, BiagioPierce, JessicaDaros, Ruan R.Pierri, FrancescaDarre, Michael J.Pierzchalska, MałgorzataDashper, KatePieszka, MagdalenaDatta, RahulPieszka, MarekDavail, BlandinePiluzza, GiannellaDavid Greg, RileyPinares Patino, CesarDavies, HelenPiñeiro, CarlosDavis, EricPinna, CarloDavis, Michael E.Pinotto, LucianoDavis, RobertPiórkowska, KatarzynaDavoli, RobertaPiotrowska-Tomala, Katarzyna KarolinaDawson, JessicaPirgozliev, Vasilde Alencar Nääs, IrenilzaPittet, FlorentDe Andrés Cara, Damián F.Pla-Aragones, LuisDe Araújo Oliveira, Chiara AlbanoPlasenzotti, Robertode Araújo, José Pedro PintoPleshakova, Tatyana O.De Briyne, NancyPlowman, JeffDe Camargo, Gregório Miguel FerreiraPlush, Kate J.De Cesare, AlessandraPoessel, Sharon A.De Clercq, DominiquePogorzelska-Nowicka, EwelinaDe Cramer, Kurt G.M.Pogrmic-Majkic, KristinaDe Jong, IngridPohl, ChristianDe Jonge, Leon LHPokharel, Sanjeeta SharmaDe Keuster, TinyPolasik, DanielDe La Fuente Vázquez, JesúsPolidori, PaoloDe La Fuente, GabrielPollo, SimoneDe Mori, BarbaraPomerantz, OriDe Olde, EvelienPongrácz, PéterDe Palo, PasqualePonte, GiovannaDe Paula, Eduardo MarosteganPonzoni, RaulDe Rensis, FabioPopescu, SilvanaDe Vries, AlbertPorto, SimonaDeAraugo, JodiPorto, Simona Maria CarmelaDębski, BogdanPotes, Maria EduardaDec, MartaPotvin, DominiqueDecaro, NicolaPourlis, ArisDed, LukasPowell, DavidDeen, JohnPozzo, LuisaDego, Oudessa KerroPrada, Christopher FernandezDegroote, JeroenPrado-Oviedo, Natalia A.Del Pino, Francisco Augusto BurkertPrearo, MarinoDelgado Bermejo, Juan VicentePresto Åkerfeldt, MagdalenaDelgado, CherylPretto, TobiaDelgado, Juan VicentePrins, Jan-BasDelgado, MikelProppe, DarrenDelibes-Mateos, MiguelProskuryakova, Anastasia A.Dell, ColleenProtopopova, AlexandraDella Casa, GiacintoProtopopova, AlexandraDella Malva, AntonellaProudfoot, KathrynDelon, NicolasProudfoot, KatyDelon, NicolasProulx, GilbertDeMello, MargoProvolo, GiorgioDemyda, SebastianPtacek, MartinDengler, FranziskaPugliese, CarolinaDennis, UchechukwuPulido, Rubén GuillermoDenwood, MatthewPulina, GiuseppeDeplazes, PeterPurwin, CezaryDescovich, KrisPutman, BreeDescovich, KrisPybus, Margo J.Detilleux, JohannQi, GuanghaiDettori, Maria LuisaQuattrocchi, FedoraDevriendt, NausikaaRabbitt, LouiseDi Cerbo, AlessandroRaboisson, DidierDi Gerlando, RosaliaRaedts, PieterDi Giancamillo, AlessiaRagusa, Maria AntoniettaDi Martino, GuidoRahmatalla, Siham A.Di Trana, AdrianaRaj, MohanDiana, AlessiaRamachandiran, IyappanDiao, QiyuRambozzi, LuisaDiaz De Cerio, OihaneRamchandani, DivyaDíaz, ClaraRamin, MohammadDickman, ChrisRamírez-Mella, MónicaDicksved, JohanRanadheera, Chaminda SenakaDieni, CristinaRandazzo, BasilioDierenfeld, EllenRandhawa, ImtiazDierick, NoëlRandhawa, ImtiazDifford, Gareth F.Randle, HayleyDillon, JasmineRandler, ChristophDing, LumingRando, AndreaDing, XiangdongRankin, Shelley C.Ditchkoff, SteveRapetti, LucaDixon, LauraRasmussen, Morten DamDixon, LauraRathgeber, BruceDo, Duy NgocRato, Luís Pedro FerreiraDo, Duy NgocRavindran, V.Dobrowolski, PiotrRawlings, John MerrittDocon, M PilarRawski, MateuszDodds, W. JeanRay, ParthaDoherty, OrlaRaynor, EdwardDomínguez Rebolledo, Álvaro EfrénRead, JohnDominguez, Juan CarlosRebollo, SalvadorDoran, OlenaRecoules, EmilieDörgeloh, WernerRedaelli, VeronicaDorszewski, PiotrReese, Laura A.Dos Santos Niculau, EdenilsonRefshauge, GordonDoughty, AmandaRegaiolli, BarbaraDoughty, AmandaRegula, JulitaDowling, SeanaReichler, Iris MargaretDraper, ChrisReifinger, MartinDrażbo, Aleksandra AlicjaReiss, MichaelDrews, BarbaraRempel, LeaDriessen, BertRempel, Lea A.Drögemüller, CordRen, LinzhuD’Souza, DarrylRen, ZhanjunDuberstein, Kylee JoRengaraj, DeivendranDubois, SaraRengaraj, DeivendranDuckett, SusanRenna, ManuelaDuda, MałgorzataRestoux, GwendalDuggan, VivienneRevilla, ManuelDukkipati, RaoReyer, HenryDumetre, AurélienReynolds, JimDumorné, KellyRibeiro, GabrielDunshea, FrankRibeiro, Karina GuimarãesDüpjan, SandraRibeiro, Leonir BuenoDurairaj, VijayRibeiro, Maria NormaDuran, CarolinaRicaud, KarineDurmic, ZoeyRichards, Bryan J.Durward-Akhurst, Sian ARichards, Elizabeth A.Duscher, Georg GerhardRico, DanielDuszka, KalinaRiedstra, Bernd J.Dutta, RahulRifu, XuDuvnjak, MarijaRighi, FedericoDyson, SueRilcheva, Violeta S.Dzidic, AlenRiley, ChristopherDziekońska, AnnaRiley, SophieE, GuangxinRipoll, GuillermoEason, PerriRivero Viera, Myriam JordanaEbensperger, GermánRiveros, José LuisEberhart-Phillips, LukeRizos, DimitriosEckelkamp, ElizabethRizzi, ChiaraEcker, SaanRoberts, JulieEda, ShigetoshiRobertson, AndyEddison, JohnRobichaud, M. VillettazEder, KlausRobinson, LaurenEdwards, Katie L.Rocchi, MarianoEdwards, Lauren E.Rochell, Samuel J.Edwards, SandraRodenburg, BasEdwards-Callaway, LilyRodger, JohnEgenvall, AgnetaRodrigues, SandraEggel, MatthiasRodríguez Alvarez, LleretnyEhrhardt, RichardRodríguez, PedroEid, NabilRodríguez-Estévez, VicenteEik, Lars OlavRoessler, ReginaEinspanier, RalfRogers, Chris W.Ekholm Fry, NinaRogers, LesleyEkker, MarcRogiewicz, AnnaEl Alami, AbderrazakRogiewicz, AnnaElbers, GereonRohrer, Gary AlanEleyadeth-Meethal, MuhammedRolinec, MichalElgendy, RamyRollin, Bernard E.Elgendy, RamyRollin, EmmanuelElguezabal, NataliaRomano, NiclaElleder, DanielRomans, Emma Fàbrega IEllingsen-Dalskau, KristianRoncarati, AlessandraEllis, Andrea DorotheaRondina, DavideElmi, AlbertoRood, Julian I.Elokil, AbdelmotalebRopka-Molik, KatarzynaElolimy, AhmedRoques, JonathanElse, Terry AnnRørtveit, RunaElsholz, SabrinaRørvang, Maria VilainElwan, HamadaRosales Nieto, CesarElwood, Robert W.Rose, PaulElzo, Mauricio A.Rosell, JoanEnders-Slegers, Marie-JoseRosell, Joan M.Endo, NatsumiRosell, Joan M.Epis, SaraRoselli, MariannaErasmus, MarisaRosen, Benjamin DErickson, Howard H.Ros-Freixedes, RogerErickson, PeterRoss, JamesErol, ErdalRoss, SteveEssen, Erica VonRossi, RaffaellaEssler, JenniferRoth, LinaEstanis, NavarroRoura, XavierEsteban, Ma. AngelesRowan, AndrewEusebi, Paulina GarcíaRowland, KayleeExbrayat, Jean-MarieRuda, AlešFaccia, MicheleRudi, KnutFaciola, AntonioRuelle, ElodieFaggio, CaterinaRufener, ChristinaFalcone, Pasquale MarcelloRugoho, InnocentFaldyna, MartinRuhnke, IsabelleFalzon, GregRumpold, BirgitFan, MingRutberg, Allen T.Fan, ZhenxinRząsa, AnnaFan, ZhiyongRząsa, AnnaFang, LingzhaoSaadeldin, IslamFanson, KerrySaastamoinen, MarkkuFaria, PaulaSáenz, LeonardoFarmer, ChantalSáez Casado, María IsabelFarmer-Dougan, ValeriSaint-Cricq, PhilippeFarnell, Morgan B.Sakkas, PanagiotisFarnell, YihuaSakomura, Nilva KazueFarnworth, MarkSalak-Johnson, JaneenFattorini, NiccolòSalak-Johnson, JaneenFaustini, MassimoSalak-Johnson, Janeen L.Favas, PauloSalas, MarinaFawcett, AnneSalas, MarinaFazio, EsterinaSalavati, MazdakFechner, HenrySalavati, MazdakFederica, PirroneSalem, Abdelfattah Z.M.Fei, Andrew Chang-YoungSalmon-Divon, MaliFeldmann, MarenSamadi, Sayyed AliFelix, AnandaSamaras, AthanasiosFels, MichaelaSamardžija, MarkoFenner, KateSamková, EvaFerenc, PajorSamolińska, WiolettaFerlazzo, AdrianaSamuel, RyanFernandez, CarlosSamy, AhmedFernández, IgnacioSan Andres, ManuelFernández-Bellon, HugoSan Juan Serrano, FuencislaFernández-Bellon, HugoSanchez, Marie-PierreFernández-Juricic, EstebanSandoe, PeterFernando Schütz, LuísSandøe, PeterFerneborg, SabineSandra, Olivier.Ferrante, ValentinaSaneyasu, TakaokiFerreira-Dias, Graça M.Sangild, Per TorpFerreras, Javier CañónSanna, FrancescaFeuerbacher, EricaSantana, Miguel H.A.Figueroa, JaimeSantini, AntonelloFilannino, PasqualeSantos, Ana SofiaFiliciotto, FrancescoSantos, F.A. AbadeFine, AubreySantos, LuanFine, MichaelSantos, Tatiana CarlescoFinelli, CarmineSaqui-Salces, MilenaFinke, Mark D.Saraiva, João L.Finlayson, GraemeSardi, LucaFioretti, AlessandroSardina, Maria TeresaFiset, SylvainSarmiento, LuisFisher, MarkSaro, CristinaFitch, JerrySarti, Francesca MariaFitzsimmons, CarolynSartori, CristinaFlorek, MariuszSato, Marcello OtakeFlythe, MichaelSatou, TadaakiFoldager, LeslieSatta, YokoFoley, Aaron M.Sattler, WolfgangFonseca, FabyanoSatué, KatiuskaFontbonne, AlainSavaglia, JemmaFontes, PedroSavell, JeffFont-i-Furnols, MariaSavvas, IoannisForeman, Anne M.Sbrissia, André FischerForkman, BjörnScandurra, AnnaForrest, RachelScandurra, CristianoFortina, RiccardoSchaefer, Allan L.Fowler, KatieSchäfer-Somi, SabineFoxcroft, George R.Schärer-Umpierre, MichelleFradinho, M.J.Schedle, KarlFradinho, Maria JoãoScheffler, JasonFraile, LorenzoScheffler, Tracy L.Fraley, Gregory S.Scherk, MargieFranciosini, MariaSchetters, TheoFranco, Nuno HenriqueSchiavone, AchilleFranczak, AnitaSchild, Sarah-Lina AagaardFranks, BeccaSchinckel, Allan PFranzoi, MarcoSchlecht, EvaFreeman, ElizabethSchletterer, MartinFregonesi, JoseSchmoelzl, SabineFreire, RafaelSchmucker, SonjaFreitas, Patrícia D.Schoeffmann, GudrunFriedman-Einat, MiriamSchoenebeck, JeffreyFriel, MarySchott, Harold C.Friendship, RobertSchroeder, KatyFröhlich, MarlenSchulte, BruceFry, LindsaySchulz, CarstenFry, LindsaySchuttler, StephanieFrynta, DanielSchwarz, LukasFthenakis, GeorgeSchwarz, TomaszFu, XingScocco, PaolaFuerst, AntonScofield, RoseFuerst-Waltl, BirgitScoley, GillianFugazza, ClaudiaScudiero, RosariaFujie, TomoyaSearcy, WilliamFujimori, KoSebastian, AimyFukuda, KenjiSecci, GiuliaFuller, GraceSeddon, Philip J.Fumerton, RichardSedlacek, RadislavGäbel, GottholdSedmikova, MarketaGabler, ChristophSegato, SeverinoGabler, Nicholas KSehgal, InderGácsi, MártaSeidavi, AlirezaGadberry, ShaneSeiquer, IsabelGagaoua, MohammedSekino, MasashiGai, FrancescoSelvaggi, MariaGalanopoulos, KonstantinosSepulveda, David R.Gálik, BranislavSeradj, Ahmad RezaGalina, Carlos S.Serrano-Montes, José LuisGalindo-Villegas, JorgeSerrano-Perez, BeatrizGall Troselj, KoraljkaServili, AriannaGallardo, María A.Sevi, AgostinoGallardo, RodrigoSeyfang, JemmaGallo, CarmenSgorbini, MicaelaGallo, LuigiSha, TaoGalvão, KlibsShaffer, Scott A.Gama, L. T.Shakeri, MajidGao, JieShanmugasundaram, RevathiGarbossa, Cesar Augusto PospissilSharbati, SoroushGarces Narro, CarlosSharma, ArvindGarcía Alvarado, José SantosSharma, ArvindGarcía García, Rosa MaríaSharp, ClaireGarcia Ispierto, IrinaSharp, TrudyGarcia Pardo, Maria De La LuzSharpe, Lynda L.Garcia, ArleneSHEATS, KatieGarcia, FélixSheen, Shyn-ShinGarcía-Gasca, Silvia AlejandraShek-Vugrovečki, AnaGarcia-Mazcorro, JoseShelomi, MatanGarcía-R, Juan CarlosShelton, DeliaGarcía-Rebollar, PalomaShen, JunshiGarcía-Rodríguez, AserShevlin, HenryGardón, Juan CarlosShi, FangxiongGarland, AlexisShi, Xin’eGarner, JosieShieh, Bao-SenGarnsworthy, PhilShim, Kwan SeobGarofalo, MariangelaShimizu, AkioGartner, MariekeShirahata, TatsuyaGaspa, GiustinoShivley, Chelsey B.Gautron, JoelShock, DanielGaworski, MarekShriver, AdamGazzano, AngeloShuai, SurongGazzano, AngeloSidiropoulou, ErasmiaGebhardt-Henrich, SabineSiegert, WolfgangGebreyesus, GrumSiegford, JaniceGengler, NicolasSiettou, ChristinaGentz, MariaSih, AndrewGeorg, HeikoSilva, Aleksandro S. DaGeorge, Kelly A.Silva, KarineGermonpré, MietjeSilvestre, Miguel AngelGhassemi Nejad, JalilSimitzis, PanagiotisGhassemi Nejad, JalilSimões, JoãoGhosh, SumitSimojoki, HeliGiannenas, IliasŠimoník, OndřejGiannetto, ClaudiaSinclair, MichelleGibson, JohannaSingh, BhupinderGibson, Troy J.Singh, ParamGiersberg, Mona FranziskaSingh, PreetGieryńska, MałgorzataSingh, RajeshGiljov, AndreySingh, YashpalGils, Hein VanSinha, AmitGiovambattista, GuillermoSiniscalchi, MarcelloGiovanardi, DavideSinski, JenniferGiráldez, Francisco JavierSiqueira, FrancieleGirdland Flink, LinusSiracusa, CarloGiri, SibSiren, Alexej P. K.Giuliotti, LorellaSirri, FedericoGiuseppe, AnnuziataSkoufos, IoannisGlade, Michael J.Skřivanová Eva, EvaGlatz, PhilŚlaska, BrygidaGlenk, Lisa MariaSlater, MargaretGlenk, Lisa MariaSlater, MargaretGodfrey, Robert W.Slater, Margaret R.Gold, JeniferSławińska, AnnaGołębiewski, MarcinŚliżewska, KatarzynaGolledge, HuwSlomczynska, MariaGómez Carpio, MayraSłupecka-Ziemilska, MonikaGoñi, Fernando R.Smetana, SergiyGonzález Martín, Juan VicenteSmith, AdrianGonzalez, JohnSmith, AndrewGonzalez, LucianoSmith, DanielGonzalez, ManuelSmith, EdGonzalez-Bulnes, AntonioSmith, GrahamGonzález-Ramírez, Mónica TeresaSmith, TomGonzalez-Redondo, PedroSmołucha, GrzegorzGonzález-Ríos, HumbertoSnelling, Warren M.Gonzalez-Rivas, PaulaSnowdon, CharlesGoodband, R.D.Sobek, ZbigniewGoodla, LavanyaSoglia, FrancescaGoodrich, ErinSole-Guitart, AlbertGoossens, KarenSolovyev, NikolayGordon, StuartSoltani, MehdiGorlov, IvanSomers, Joris R.Gort, AraceliSommerfeld, VeraGoto, KenichiSong, JeongminGoto, TatsuhikoSong, TongxingGradinaru, Andrei CristianSørensen, Dorte BratboGraham, Melanie L.Sossidou, EvangeliaGrandin, TempleSossidou, Evangelia N.Granier, SophieSovrano, Valeria AnnaGrashuis, JasperSpadola, FilippoGrau, CarlosSpannring, ReingardGray, HelenSparrey, JulianGreco, BrianSpence, KelseyGreef, Jörg Michael GreefSpengler Neff, AnetGreen, ClaySperoni, MarisannaGreen, Jeffrey D.Spielman, DerekGreen, Ronald MichaelSpiers, DonGreenwood, Emma C.Spindler, BirgitGreer, AlanSpinka, MarekGrela, Eugeniusz RyszardSpinu, MarinaGriffiths, CharlesSpörndly, EvaGrignolio, StefanoSpringer, SvenjaGrigorakis, KrytonSpruell, PaulGrilli, EsterSquires, E.J.Grissett, GretchenStacey, M.Grochowska, EwaStachowiak, MonikaGrondin, SimonStádník, LuděkGroße Beilage, ElisabethStafleu, FransGroves, PeterStafuzza, Nedenia BonvinoGryz, JakubStapleton, PhoebeGryzińska, Magdalena MariaStatham, PoppyGrześkowiak, ŁukaszStear, MichaelGrześkowiak, Łukasz M.Stefania, PucciarelliGrzybek, MaciejStefaniuk-Szmukier, MonikaGuan, WutaiStefaniuk-Szmukier, MonikaGuan, YongjuanStein, HansGuay, KimberlySteinbach, ChristophGuay, Patrick-JeanSteinberg, Eliana RuthGuelfi, GabriellaSteinhagen, DieterGuerin, FrançoisSteinman, AmirGunia, MelanieStella, JudithGunnarson, StefanStenzel, LynneGüntürkün, OnurStephan, WildeusGuo, KaijunStephen, O’GradyGurgul, ArturStephens, DiGutiérrez, Ana MaríaStephens, TanyaGutierrez, JuanSter, CélineGutiérrez-Martin, César B.Stetina, Birgit U.Guy, JonathanSteup, MartinGuzhva, OleksiyStocco, CarlosGuzmán-Pino, SergioStock, Friederike KatharinaH. Eisemann, JoanStork, Christian J.Ha, JamesStrillacci, Maria GiuseppinaHabus, JosipaStringham, OliverHać-Szymańczuk, ElżbietaStrive, TanjaHadaš, ZdeněkStrube, Mikael LenzHadley, PhilStruthers, SarahHaenlein, George F. W.Stubbs, ChristopherHagen, JennyStumpff, FriederikeHairgrove, ThomasSturaro, EnricoHalama, JonathanSturgill, Tracy L.Halas, VeronikaSu, HuaweiHaley, Nicholas J.Su, HuaweiHall, KatieSu, YongHall, RobynSuarez-Vega, AroaHallerman, EricSudano, Mateus J.Hammerl, Jens AndreSugaya, TakumaHampton, JordanSugita, HaruoHan, FelicitySuk Chun, FungHan, XuefengSultan, KhaledHan, ZhaoyuSuman, Surendranath P.Handl, StefanieSummer, AndreaHanne, HansenSummers, KimHänninen, LauraSun, DongboHannon, Kevin M.Sun, LvhuiHanot, PaulineSun, XuezhaoHansen, Tia G. B.Sun, YongfengHanusová, EmíliaSung, Kyung-illHanzawa, NaotoSuperchi, PaolaHardefeldt, LauraSurai, Peter F.Hardouin, EmilieSutherland, MhairiHarris, Lauren K.Svoboda, MartinHarrison, AdrianSwaggerty, ChristinaHart, SteveSwanson, KendallHartnack, SonjaSwelum, AymanHarvey, NaomiSwerczek, ThomasHarvey, SimonSwerczek, Thomas W.Hashiguchi, YasuyukiŚwięch, EwaHaskell, MarieŚwitoński, MarekHässig, MichaelSylte, Matthew J.Hattori, KaoruSymons, JenniferHavelka, MilosSzabo, JuditHawkins, Emma L.Szczepanek, JoannaHawkins, PennySzczerbinska, DanutaHay, El HamidiSzmatoła, TomaszHayashi, ShinichiroT. Cenci-Goga, BeniaminoHayes, MorganT. Cenci-Goga, BeniaminoHayirli, ArmaganTabata, RyoichiHazel, SusanTadich, TamaraHeadon, Denis JTagang, AluwongHeberlein, Thomas A.Tahamtani, FernandaHediger, KarinTajchman, KatarzynaHeerkens, JasperTajima, YukoHeiderstadt, KathleenTakahashi, SumioHeins, BradleyTakahashi-Omoe, HiromiHeleski, CamieTakehiro, NishidaHellwing, Anne Louise FrydendahlTakeshi, TsukaHemingway, AnnTalamini, EdsonHemsworth, Lauren M.Tallentire, CraigHendricks, Joan C.Tallo-Parra, OriolHennessy, DeirdreTamura, HidekiHenry, DarrenTan, BieHenry, ErinTan, Jean-YinHerbut, PiotrTang, ShiyuHernández, FuensantaTang, ZhonglinHernandez-Gomez, ObedTanui, Collins K.Heroldová, MartaTarantola, MartinaHerrera Haro, José GuadalupeTassone, SoniaHerrick, Jason R.Taus, NaomiHerring, AndyTaylor, PetaHerring, Andy D.Taylor, PetaHerskin, Mette S.Te Pas, MarinusHertel, AnneTeixeira, CarlosHervás, GonzaloTellez-Isaias, GuillermoHervera, MartaTerayama, HayatoHess, ClaudiaTernman, EmmaHess, StevenTetens, JensHeuvelink, AnnetTettamanti, GianlucaHiby, EllyTheuerkauf, JörnHickman, DebraThodberg, KarenHillmann, EdnaThomas, DavidHiney, KrisThomson, DanHirase, ShotaroThomson, JackHoagwood, KimberlyThomson, JenniferHobbs, Sarah JaneThöne-Reineke, ChristaHockenhull, JoThonhauser, GerhardHoffman, RhondaThorne, Mark S.Hoffmann, GundulaThorup, FlemmingHokkanen, Ann-HelenaTian, QiyuHolland, Katrina E.Tiezzi, FrancescoHollin, GregTinkler, StacyHolloway, StevenTiplady, CatherineHolmquist, JeffTlili, SarraHolst, Bodil StrömTobias, EdwardHolt, JonTodaro, MassimoHolzhauer, MennoTodde, GiuseppeHong, Bo-YoungTokach, MikeHong, Cheng-YihTokarska, MałgorzataHood, JenniferTokuda, MasaharuHopkins, David L.Tolhurst, Bryony A.Horback, KristinaTomaszewska, EwaHorohov, DavidTomažič, IztokHorta, OscarTomczyk, ŁukaszHoste, HerveToms, DerekHotea, IonelaTopál, JózsefHötzel, MariaTorres, AlexandrHou, FujiangTrabalza-Marinucci, MassimoHoude, PeterTrabalza-Marinucci, MassimoHoupt, Katherine AlbroTresoldi, GrazyneHowell, TiffaniTrevarthen, AnnaHowie, MikaelaTrocino, AngelaHoy, SteffenTruzzi, CristinaHtoo, JohnTryjanowski, PiotrHu, JianhongTsepilov, YakovHu, ZhiyongTsouloufi, Theodora K.Huang, FeiruoTsukahara, TakamitsuHuang, JiangengTsuruta, ShogoHuang, Li-TungTu, YanHuang, San-YuanTudisco, RaffaellaHuang, Wu-JangTufarelli, VincenzoHuggenberger, StefanTufarelli, VincenzoHughes, KrisTulli, FrancescaHuntingford, JaniceTullo, EmanuelaHuntington, Gerald B.Tumova, EvaHuo, LijunTun, Hein MinHurford, CliveTunnicliffe, Sue DaleHurley, Karinna B.Turchi, BarbaraHurley, Walter LTurk, RomanaHuth, MartinTurner, Patricia V.Hutton, VickiTurner, SimonHyun, Sang-HwanTuśnio, AnnaIaffaldano, NicolaiaTuz, RyszardIanni, AndreaTydén, EvaIbelli, AdrianaTynes, ValarieIchihashi, HidekiUccheddu, StefaniaIchinohe, ToshiyoshiUchida, KentaIdrus, ZulkifliUlusoy, BeyzaIjichi, CarrieUni, ZehavaIkeda, DaisukeUpadhaya, SantiInfascelli, FedericoUrschel, KristineInfascelli, FedericoUsmani, AbulIngham, Aaron B.Uwiera, RichardIris, RibitschUwiera, RichardIrvine, LeslieUyeno, YutakaIshikawa, DaitaroVaarst, MetteIslam, Md. NahidulVacca, Giuseppe MassimoIson, Sarah H.Vaiman, DanielIstván, NagyValasi, IreneIvanov, Yuri D.Valenzuela, CarolinaIyer, ChitraValle, EmanuelaIzeta, AnderVallée, EmilieJackson, PatVallortigara, GeorgioJackson, Samuel P.Van Amburgh, MichaelJacob, RobinVan De Vis, HansJacobs, LeonieVan De Weerd, HeleenJalongo, Mary RenckVan Den Berg, IreneJang, Jae CheolVan Den Broeck, WimJang, Young DalVan Der Aar, PietJankowski, JanVan Der Pol, Carla W.Jansen, WiebkeVan Der Saag, DominiqueJaworska, DanutaVan Der Werf, JuliusJaworski, NeilVan Eerdenburg, FrankJenks, Jonathan A.Van Emon, MeganJensen, Annette NygaardVan Erp-van Der Kooij, LennyJensen, Rasmus BovbjergVan Krimpen, MarinusJerez, SalvadorVan Niekerk, TheaJeyanathan, Jeyamalarvan Staaveren, NienkeJeyaruban, M. G.Van Uhm, DaanJeżewska-Frąckowiak, JoannaVan Vollenhoven, ElizeJiang, LiliVan Wettere, WilliamJiang, XunpingVanderschuren, L.J.M.J. (Louk)Jirkof, PaulinVanessa, DazukJobling, MalcolmVargas-Bello Perez, EinarJohannes, BerndVasconcelos, Ana Carina NogueiraJohnson, AmyVasconi, MauroJohnson, EmmaVasseur, E.Johnson, Jay S.Vaughn, Mathew.A.Johnson, JoshuaVecchiato, Carla GiudittaJohnson, RebeccaVeerasamy, SejianJohnsson, MartinVelazquez, MiguelJohnston, LeeVeldkamp, TeunJonas, ElisabethVelkers, FranciscaJongman, EllenVenail, PatrickJordana, JordiVendramin, NiccolòJorge, ErikaVeneziani, MarioJoshi, ChetanchandraVentrella, DomenicoJoy, MargalidaVentura, Beth A.Józefiak, DamianVerdon, MeganJúnior, Dorgival M De LimaVernes, KarlJuškienė, VioletaVerruck, SilvaniKabir, S. M. LutfulVerstegen, John P.Kaczmarek, Monika M.Vervuert, IngridKaczmarek, SebastianVeskoukis, Aristidis S.Kaczmarek, SebastianVeskoukis, Aristidis S.Kaji, KoichiViegas, João CarlosKakinuma, MikiVigors, BelindaKamler, Jan F.Vigors, StaffordKandil, Omnia M.Vihervaara, AnniinaKANEDA, TakeharuVilar Ares, Maria JKang, HuiminVilar, Jose ManuelKang, JinheVillarruel - López, AngélicaKaraca, SerhatVinchurkar, SamirKaramon, JacekVisker, MarleenKaranikolas, PavlosVisscher, ChristianKaratzia, Maria-AnastasiaVisser, CarinaKaravoltsos, SotiriosVisseren-Hamakers, Ingrid J.Kareskoski, MariaVitali, AndreaKarpiński, MirosławVitali, MarikaKasarda, RadovanVizzarri, FrancescoKatayama, KentaroVogeler, Colette S.Katayama, YukitoshiVolsche, ShellyKatsoris, PanagiotisVon Meyer-Hoefer, MarieKaukonen, EijaVonk, JenniferKawamura, HiroakiVoy, BrynnKawasaki, KiyonoriVrbanac, ZoranKazeto, YukinoriVrentas, Catherine E.Keates, HelenVyas, DiwakarKedrowicz, AprilWaal, Theo DeKeegan, JasonWagener, KarenKehoe, SylviaWagley, SariqaKeim, Juan PabloWakshlag, JosephKelley Wamsley, KelleyWaleron, KrzysztofKelley, Dale E.Walker, PaulKells, NikkiWall, HelenaKemper, NicoleWallis, LisaKemski, MeganWang, BingKendall, Holli A.Wang, ChaoKenéz, ÁkosWang, FangKennedy, UttaraWang, FenglaiKeogh, KateWang, GuitangKerouanton, AnnaëlleWang, HaifengKerr, Caroline AudreyWang, JianhuaKetta, MohamedWang, JiwenKhamas, WaelWang, MengzhiKhan, AjmalWang, ShiKhattak, FarinaWang, Shu-YinKhoddami, AliWang, SongboKholodov, VladimirWang, XiKhoo, ShaunWang, Xiao-LongKiczorowska, BożenaWang, XiuqiKiczorowska, BożenaWang, YachunKiddie, JennaWang, YuxiKierończyk, BartoszWang, ZhengchaoKikusato, MotoiWang, ZhishengKikusui, TakefumiWang, ZhiyueKil, Dong Y.Ward, AlisonKiley-Worthington, MartheWargelius, AnnaKilroy, DavidWarren, L. K.Kim, Beob GyunWarwick, CliffordKim, Beob GyunWarwick, CliffordKim, Byoung SikWascher, Claudia A. F.Kim, Dal YoungWatanabe, ShigeruKim, Hyeun BumWaterhouse, TonyKim, InhoWatson, AndreaKim, Min JungWay, JonathanKim, SungwonWeaver, AliceKind, Karen L.Webster, JohnKing, Sarah R.B.Webster, JohnKirkpatrick, BrianWebster, KoaKirkwood, Roy NevilleWeese, ScottKirnan, JeanWeger, StefanKis, AnnaWei, HongkuiKittelsen, Käthe EliseWeiler, UlrikeKjaer, JoergenWeimer, Shawna L.Kjos, Nils PetterWelbourne, Dustin JKleinhans, BelindaWelling, MickKlevenhusen, FenjaWemelsfelder, FrancoiseKnierim, UteWen, JieKnight, JimWenk, CasparKnight, PeterWeston, Jennifer F.Koda, NaokoWeston, M.Kogan, LoriWeston, Michael AKogut, MichaelWestreicher-Kristen, EdwinKogut, Michael H.Westreicher-Kristen, EdwinKoh, Cho YeowWhite, Amelia G.Koh, Ho-JinWhite, Angela M.Koide, TetsuyaWhite, GavinKokoszyński, DariuszWhite, John KerrKokoszyński, Professor DariuszWhitehouse, JamieKokou, FotiniWhitehouse, NancyKokou, FotiniWhiting, TerryKoliesnik, IevgenWhittaker, AlexandraKoltes, DawnWhitton, ChrisKoluman-Darcan, NazanWichert, BrigittaKomaki, ShoheiWick, MacdonaldKomine, HirotakaWickersham, TryonKomissarov, AlekseyWigham, EllieKong, Il-KeunWilk, IzabelaKong, XiangfengWilliams, B.M.Konieczka, PawełWilliams, Charlotte AmdiKopp, StevenWilliams, E. MarkKorwin-Kossakowska, AgnieszkaWilliams, EllenKorytar, TomasWilliams, HowelKoseniuk, AnnaWilliams, JaneKoski, SonjaWilliams, Jane M.Kotsampasi, BasilikiWilliams, LeahKouakou, BrouWilliams, PrysorKoutsos, LizWilliams, RichardKowalczyk, AlicjaWilliams, SamKowalska-Góralska, MonikaWills, AlisonKowalski, Radoslaw KajetanWilson, GeorgeKowalski, Zygmunt MaciejWilson, JuliaKozdru, WojciechWilson, MeganKozłowski, KrzysztofWilson, VanessaKrakowski, LeszekWilson-Frank, Christina R.Kral, AndrejWimbush, KarenKralik, ZlataWindisch, WilhelmKrause, TobiasWindisch, WilhelmKrauze-Gryz, DagnyWiniarska-Mieczan, AnnaKrawzel, PeterWinter, CarolineKrebs, Bethany L.Wiseman, M.Krebs, GayeWitmer, GaryKreisler, RachaelWojdak-Maksymiec, KatarzynaKreuzer, MichaelWójtowski, JacekKröger, SusanWojtysiak, DorotaKról, JolantaWolf, PeterKróliczewska, BożenaWolf, PetraKronberg, ScottWolf, Tanja EKubinyi, EnikoWon, SeunggunKues, Wilfried A.Wong, TentsaoKuhne, FranziskaWood, KevinKumar, SanathWoodworth, Jason C.Kumar, SanjayWormald, DennisKupczyński, RobertWróbel, BarbaraKurasiak-Popowska, DanutaWronski, TorstenKusano, KanichiWu, ShengruKvidera, SaraWu, WangjunKwon, Young M.Wu, XinLadewig, JanWu, XinshengLafferty, DianaWu, ZhonghongLaFollette, Megan R.Wurtz, KailtinLahbib-Mansais, YvetteXu, Ling-YangLahdenpera, MirkkaXu, TianjianLai, ChanghuaXu, XuewenLalander, CeciliaYamamoto, TakeshiLaliotis, George PYamamoto, ToshiharuLalmanach, Anne-ChristineYamashita, MichiakiLammi, Mikko J.Yamauchi, HiroyukiLamminen, MarjukkaYan, PingLan, XianyongYan, XuLan, XianyongYang, Cheng-YaoLandfried, Lauren K.Yang, LingLandi, VincenzoYang, LiuLangendijk, PieterYang, QienLanzetti, AgneseYang, QiyuanLara, Leonardo José CamargosYang, Run-jun YangLarsen, JenniferYang, Shyi-KuenLarsen, Mona Lilian VestbjergYang, WucaiLasagna, EmilianoYang, ZongbaoLaskowski, ŁukaszYao, JunhuLassala, Arantzatzu L.Yao, JunhuLatorre, Juan DavidYao, WenLatorre, Maria AngelesYasuike, MotoshigeLau, QuintinYayota, MasatoLaudadio, VitoYeung, PollyLauriault, LeonardYiakoulaki, MariaLaven, LindaYilmaz, SevdanLaven, RichardYin, WenqingLavon, YanivYngvesson, JennyLavrenčič, AndrejYokobori, NoemíLawrence, ChristianYokoyama, SaichiroLay, DonYonekura, ShinichiLazarowski, LuciaYoo, KeunjeLe Bon, MelanieYoon, Sang-PilLean, IanYosef, ReuvenLeavesley, DavidYou, XinxinLederman, ZoharYoung, JulieLee, ChanheeYoussef, Fatma Mohamed AhmedLee, Hong-GuYu, Yu-HsiangLee, Kyung-WooYurchenko, Andrey A.Lee, Mark AYves, NysLee, MichaelZaefarian, FifiLee, Seung-HwanZahan, MariusLee, Tzu-TaiZak, LouisaLee, YongseokZaleski, HalinaLefaucheur, LouisZamaratskaia, GaliaLefaucheur, LouisZambonelli, PaoloLegge, Melissa MarieZampiga, MarcoLegge, Sarah M.Zanine, Anderson De MouraLei, ChuzhaoZanzani, SergioLeitão, AlexandreZapico, Sara CasadoLePage, BenZappaterra, MartinaLepczyk, ChristopherZdenek, VolekLepczynski, AdamZeitz, JohannaLeroux, ChristineZeng, QiufengLeroux, Louis-PhilippeZentek, JuergenLessard, MartinZhang, GenxiLessire, MichelZhang, GenxiLetek, MichalZhang, HaijunLétourneau-Montminy, Marie-PierreZhang, HepingLevart, AlenkaZhang, HongpingLevesque, CrystalZhang, KeyingLevionnois, OlivierZhang, NaifengLevrino, Gustavo MaríaZhang, ShihaiLevy, Julie K.Zhang, YingJunLewejohann, LarsZhang, YushengLewis, EvaZhang, ZheLewis, HelenZhao, XingboLey, Jacqueline MZheng, AijuanLi, AnningZheng, WeichaoLi, BaomingZhou, BoLi, ChangxiZhou, LeiLi, DiyanZhou, XihongLi, Dr. ZijianZhou, YangLi, FengeZhou, YanminLi, JinyaoZhou, ZhenqiLi, JunyaZhuo, ZhuLi, LijiaZielak-Steciwko, AnnaLi, MaZinn, R.A.Li, MengmengZivotofsky, AriLi, Prof. JiakuiZobel, GosiaLi, QiulingZowalaty, Mohamed E. ElLi, ShijunZsolnai, AttilaLi, SidongZsolt, MaticsLi, SufenZsuzsanna, BőszeLi, WeifenZucca, EnricaLi, XinweiZuolo, FedericoLi, XinyunZupan, ManjaLi, YangZwierzchowski, LechLi, Yihang


